# Supplementary material for: Perspectives in machine learning for wildlife conservation
Source: Nat Commun. 2022 Feb 9;13:792. doi: 10.1038/s41467-022-27980-y (PMC8828720; doi:10.1038/s41467-022-27980-y)
Supplement: Supplementary file 1 — Supplementary Information [file 41467_2022_27980_MOESM1_ESM.pdf]

Perspectives in machine learning for wildlife conservation

Devis Tuia[1]* Benjamin Kellenberger[1]* Sara Beery[2]*
Blair R. Costelloe[3,4,5]* Silvia Zuffi[6] Benjamin Risse[7]
Alexander Mathis[8] Mackenzie W. Mathis[8] Frank van Langevelde[9]
Tilo Burghardt[10] Roland Kays[11,12] Holger Klinck[13]
Martin Wikelski[3,4] Iain D. Couzin[3,4,5] Grant van Horn[13]
Margaret C. Crofoot[3,4,5] Charles V. Stewart[14] Tanya Berger-Wolf[15]

November 30, 2021

1. School of Architecture, Civil and Environmental Engineering, Ecole Polytechnique Fédérale de Lausanne (EPFL), Switzerland
2. Department of Computing and Mathematical Sciences, California Institute of Technology (Caltech), United States of America
3. Max Planck Institute of Animal Behavior, Germany
4. Centre for the Advanced Study of Collective Behaviour, University of Konstanz, Germany
5. Department of Biology, University of Konstanz, Germany
6. Institute for Applied Mathematics and Information Technologies, IMATI-CNR, Italy
7. Computer Science Department, University of Münster, Germany
8. School of Life Sciences, Ecole Polytechnique Fédérale de Lausanne (EPFL), Switzerland
9. Environmental Sciences Group, Wageningen University, Netherlands
10. Computer Science Department, University of Bristol, United Kingdom
11. Department of Forestry and Environmental Resources, North Carolina State University, United States of America
12. North Carolina Museum of Natural Sciences, United States of America
13. Cornell Lab of Ornithology, Cornell University, United States of America
14. Department of Computer Science, Rensselaer Polytechnic Institute, United States of America
15. Translational Data Analytics Institute, The Ohio State University, United States of America

* These authors contributed equally: D. Tuia, B. Kellenberger, S. Beery, B. R. Costelloe

Supplementary Information

ML term	Definition
Artificial Intelligence (AI)	The concept of a machine being able to perform higher-level, semantic reasoning.
Big data	Many definitions exist [1], but we cast "big data" as *information content for analyses whose volumes are too large to handle for users with conventional hardware.* Many sensors addressed produce "big data", in particular remote sensing, social media and camera trap networks. Analysis of such volumes of data quickly becomes intractable for conventional ML methods, in particular if the study area of interest exceeds regional ecosystems.
Classification	Assigning an entire image or video to a single category.
Computer Vision	Performing image manipulation and understanding tasks with a machine, oftentimes involving ML.
Convolutional Neural Network (CNN)	Deep learning models that contain at least one convolution layer. In such layers, neurons are organized into banks of filters that are convolved with the inputs (*i.e.*, the same filter weights are applied across multiple locations in the image). This allows reducing the number of required neurons while also providing a limited amount of translation invariance.
Data science	Like "big data", "data science" is a less-well-defined term, denoted here as an inter- or multidisciplinary research field on automated information extraction from observations or other content sources.
Deep learning	Family of prediction models that consist of neurons, grouped into three or more sequential layers, where each neuron receives the output from one (or more) previous neurons and itself predicts an output, consisting of weighted combinations of its inputs.
(visual) Descriptor	Higher-level statistics extracted from data that are supposed to summarize, or pronounce, more abstract differences within the data point to facilitate the task of the subsequent ML model, also called "feature". For example, a common descriptor used in traditional vegetation mapping on remote sensing imagery is the Normalized Difference Vegetation Index (NDVI), whose values are highly contrastive between vegetated and non-vegetated areas than bare pixel values alone. Traditional ML algorithms require manual definition and calculation of such features, whereas deep learning methods learn them automatically in the training process.
Detection	localizing the area within an image that corresponds to a category of interest, usually represented by a rectangular "bounding box" – the tightest box that could be drawn around that object while still containing all of its pixels.
Domain Adaptation	Methods to describe, evaluate, and/or tackle the challenge of out-of-domain data.
Detection rate	See "recall".
False positive	Incorrect prediction of a data point, object, or background area (*e.g.* in an image) as a certain class.
Feature	See "(visual) Descriptor".
Fine-grained classification	Label classes are denoted as "fine-grained" if they belong to a common supercategory (*e.g.*, "American Robin" and "Guineafowl" both belong to the supercategory "bird"). Fine-grained classification can be challenging if categories exhibit similar visual properties.
Individual identification	Recognizing unique instances of an object in an image or video (frame). Individual identification is usually performed through recognizing of unique visual cues that serve as "fingerprints" for an individual, such as the striping pattern of zebra or dot pattern on the back of whale shark individuals.
Inference	The act of performing prediction with a (trained) ML model.
Instance Segmentation	Grouping every pixel in an image with the other pixels corresponding to that same *instance* or object. If the image contained seven lions, each lion would be categorized with a different pixel label, even if the lions' pixel masks touch each other.
Localization	Identifying the position of an object within an image or video (frame). Unlike Detection, localization may not always include estimation of the full extents of an object, *e.g.* through a bounding box, but might be limited to spatial coordinates of the object's center.
Loss function	Numerical criterion that measures the disagreement between an ML model prediction and the Ground Truth labels. For example, the *cross-entropy loss function* returns the negative log likelihood between a predicted model probability and the label class.

Machine Learning (ML)	The ability of a computer to perform prediction tasks by learning from data (*i.e.*, without primarily relying on hard-coded cascades of rules).
Semantic Segmentation	Assigning every pixel in an image to a specific class, *i.e.*, all "lion pixels" would be labeled as such, regardless of the actual individual they belong to.
Semi-supervised learning	Training an ML model on data for which only a small subset contains labels.
Supervised learning	Training an ML model on data that consists of inputs (*e.g.*, images) and labels (*e.g.*, species names, bounding boxes).
Object detection	See "Detection".
Open-set	Scenario where a dataset may exhibit categories at test time that were unseen during ML model training. For example, a model for individual identification may be presented with images of an individual that got newly introduced to the area after training, and needs to be able to recognize it as a new individual accordingly.
Out-of-domain	Data that is not drawn from the identical set that an ML model was trained on. A good example of this would be images from a camera trap that was not seen during training.
Overfitting	Training an ML model to achieve (near-) perfect accuracy on the training set, but unacceptable accuracy on the validation or test set. Overfitting can occur if the model has too many free parameters or if the training set is not representative enough. See also "Underfitting".
Pose Estimation	2D: predicting the pixel location of known parts of an object, for example, localizing the nose, eyes, joints, and tail of a lion. 3D: predicting the parts location in space, or predicting the 3D rotation of an articulated animal skeleton.
Posture Estimation	See "Pose Estimation".
Precision	Class-wise measure of exactness of ML model predictions. A precision of 1.0 means that every prediction made by a model is correct, while one approaching 0.0 means that there is a high number of wrong predictions (see "false positive").
Recall	Class-wise measure of completeness of ML model predictions. A recall of 1.0 means that every data point with a given true label class has been correctly predicted as such by the model, while a recall of 0.0 means that the model has missed all data points of that class.
Tracking	Localizing individual objects and correctly match them between frames throughout a video or temporal sequence of images.
Training	Altering the free (learnable) parameters of an ML model to optimize it to the training dataset, usually performed by minimizing values of a Loss function.
Underfitting	An ML model underfits the training set if it cannot appropriately capture the data distribution, resulting in unacceptable accuracy. Underfitting usually occurs if the model does not have a sufficient number of free parameters. See also "Overfitting".
Unsupervised learning	Training an ML model on data that only consists of inputs, but not of labels.

Supplementary Table 1: Glossary on the most important Machine Learning (ML) terms used in this article

Model	Description	Output	Advantages	Limitations
Traditional machine learning models				
Bayesian estimation	Maximum a posteriori estimation of predictions; data are assumed to be drawn from an a priori known ("prior") distribution	Classification, regression	Can include prior knowledge about data distribution	Hyperparameter tuning can be expensive, performance depends on quality of features
Decision tree	Iterative binary split of data points according to input variables or features	Classification, regression	Very simple, intuitive and interpretable model, split thresholds can be learned from data or manually defined	Highly prone to overfitting under too many splits (large tree depth); weak performance and poor generalization capabilities if single tree (see Random Forest below); does not provide probability measures
Random Forest [2]	Ensemble of decision trees, with each tree receiving a randomized subset of data points and variables to operate on	Classification, regression	Requires little training data, can model non-linear relationships by design	Limited scalability, performance depends on quality of features
Support Vector Machine [3]	Binary classifier based on maximum margin theory	Classification, regression	Requires very little training data	Binary predictions only in original formulation; can only model non-linear relationships through kernels; performance depends on quality of features
Deep learning models				
Artificial Neural Network (ANN)	Model that applies a sequence of layers, each composed of neurons that receive all values of a data point (first layer) or outputs of the previous layer and calculate a weighted and biased combination as an output.	Classification, regression	Universal approximator, can reproduce very nonlinear behavior	Poor scalability to large data points like images; overfitting and need for early stopping in training
Convolutional Neural Network (CNN [4])	Form of ANN with convolution operators and generally large number of layers	Arbitrary (classification, regression, segmentation, mixtures, *etc.*)	Excellent performance in most machine learning tasks; high versatility	Computationally expensive; generally requires large amounts of training data
Vision Transformers [5]	Most recent alternative to CNNs that replaces convolutional layers with spatial attention modules	Arbitrary	Extremely high performance in some tasks	Extremely high computational requirements; recent method with research still ongoing
Recurrent Neural Network (RNN)	Form of ANN that ingests time series data in a pointwise manner, with each output (intermediate or final) depending on the current input as well as the previous output. RNNs can also be convolutional.	Arbitrary, on time series	Excellent performance in most machine learning tasks; high versatility	Computationally expensive; generally requires large amounts of training data; signals at early time steps in long time series may get lost in plain RNNs
Long Short-Term Memory (LSTM [6]) and Gated Recurrent Unit (GRU [7])	Form of RNN with dedicated "gates" that learn to memorize relevant signals in a time series	Arbitrary, on time series	Excellent performance in particular for long time series data	Computationally expensive; generally requires large amounts of training data

Supplementary Table 2: Most common ML models

References

[1] De Mauro, A., Greco, M. & Grimaldi, M. A formal definition of big data based on its essential features. *Library Review* (2016).

[2] Breiman, L. Random forests. *Machine learning* **45**, 5–32 (2001).

[3] Cortes, C. & Vapnik, V. Support-vector networks. *Machine learning* **20**, 273–297 (1995).

[4] LeCun, Y., Bengio, Y. & Hinton, G. Deep learning. *Nature* **521**, 436–444 (2015).

[5] Dosovitskiy, A. *et al.* An image is worth 16x16 words: Transformers for image recognition at scale. *arXiv preprint arXiv:2010.11929* (2020).

[6] Hochreiter, S. & Schmidhuber, J. Long short-term memory. *Neural computation* **9**, 1735–1780 (1997).

[7] Cho, K. *et al.* Learning phrase representations using rnn encoder-decoder for statistical machine translation. *arXiv preprint arXiv:1406.1078* (2014).
